# A descriptive study of stroke types, risk factors, clinical features, and outcomes in a tertiary hospital in Myanmar

**DOI:** 10.1186/s41182-024-00592-6

**Published:** 2024-03-18

**Authors:** Thant Zin Tun, Su Myat Han, Kazuhiko Moji, Mitsuaki Matsui

**Affiliations:** 1 Department of Global Health, Nagasaki University School of Tropical Medicine and Global Health, Nagasaki, Japan; 2 Graduate School of Biomedical Sciences, Nagasaki University, Nagasaki, Japan; 3 Department of Infectious Disease Epidemiology, Faculty of Epidemiology and Population Health, London School of Hygiene and Tropical Medicine, London, UK; 4 Department of Public Health, Kobe University Graduate School of Health Sciences, Kobe, Japan; 5 Present Address: Department of Protozoology, Institute of Tropical Medicine, Nagasaki University, Nagasaki, Japan; 6 National Centre for Infectious Diseases, 16 Jalan Tan Tock Seng, Singapore, 308442 Singapore

**Keywords:** Stroke, Hypertension, Myanmar, Risk factors of stroke

## Abstract

**Background:**

Stroke is a leading cause of death in the world, and the burden of stroke is higher in low- and middle-income countries. Understanding the risk factors, complications, and outcomes of stroke are useful for healthcare planning and resource allocation. Little information on stroke is available for many low- and middle-income Asian countries; including Myanmar, which is the focus of this study.

**Methods:**

A review was conducted of medical records for stroke admissions during 2017 in a tertiary hospital in Myanmar. The final diagnoses, risk factors, clinical features, complications, and outcomes were systematically collected from computer- and paper-based medical records.

**Results:**

Of 908 cases analysed, haemorrhagic stroke was the most common type (49%), followed by ischaemic stroke (43%). Unimproved cases were 32%. Identified risk factors of unimproved cases were ‘haemorrhagic stroke’ [adjusted odds ratio (aOR): 1.73], ‘having fever during hospitalization’ [aOR: 2.49], ‘Glasgow Coma Scale (GCS) at the admission between 9 and 14’ [aOR: 4.33], and GCS less than 9 [aOR: 42.86].

**Conclusion:**

This study is based on hospital medical records to assess stroke types, risk factors, clinical features, and outcomes in a tertiary hospital, in Nay Pyi Daw, Myanmar. The findings indicated that early case admission, improved hospital care management, and increased awareness of the modifiable risk factors within populations are crucial for preventing stroke incidents.

**Supplementary Information:**

The online version contains supplementary material available at 10.1186/s41182-024-00592-6.

## Background

Stroke is characterized by the sudden onset of focal neurological function loss due to infarction or haemorrhage in the central nervous system, with symptoms lasting more than 24 h or leading to death [1]. Stroke occurs when the blood supply to the brain is interrupted locally, and leads to a compromise in the brain’s functionality [2]. Despite the progress of stroke prevention and treatment, it remains a major health problem in low- and middle-income countries (LMICs). Although the number of deaths caused by stroke decreased in high-income countries from the years 2010 to 2019, it increased in LMICs [3]. Stroke is the fourth and second leading cause of deaths in low- and middle-income countries, respectively [3]. The disease burden of stroke in Myanmar is more significant, and in 2019 stroke was the primary cause of death in both females and males [4]. Of the ten ASEAN countries, Myanmar is the second lowest ranked with respect to age-sex standardized mortality and disability-adjusted life-years (DALYs) lost due to stroke [5].

Epidemiological studies on the prevalence of stroke, and its associated factors enable us to understand the disease epidemiology and to formulate prevention strategies. The risk factors can be categorized broadly into non-modifiable and modifiable factors. Non-modifiable factors include age, gender, race, ethnicity, and heredity; while modifiable risk factors include life-styles such as cigarette smoking and physical inactivity, and non-communicable diseases such as hypertension, dyslipidaemia, diabetes, and cardiac disorders [7–9]. Studies on risk factors related to strokes are lacking in resource-limited settings such as in Myanmar. Therefore, this study aims to assess the stroke types, risk factors, clinical features and outcomes of stroke patients admitted to a prominent referral hospital in Myanmar.

## Methods

### Study site, setting and population

This study was conducted in 1000 Bedded General Hospital, in the capital city Nay Pyi Taw, Myanmar. It is one of the highest-level referral hospitals in Myanmar, and the only hospital in Nay Pyi Taw having neurology and neurosurgical units. Cases admitted to the hospital were retrospectively identified the cases admitted to the hospital by reviewing the hospital medical records for International Classification of Diseases (tenth revision, ICD-10); and coded for stroke type (I60: non-traumatic subarachnoid haemorrhagic; I61: non-traumatic intracerebral haemorrhagic; I62: other and unspecified intracranial haemorrhagic; I63: cerebral infarction; I64: stroke, not specified as haemorrhagic or infarction; and I69: sequelae of cerebrovascular diseases). All patients diagnosed with stroke which were admitted to the hospital between 1st January and 31st December 2017 were eligible for this study.

### Data collection

Data extraction was conducted in two stages. First, the following data were extracted from the Computer Assisted Medical Record System (CAMRS) database: age, sex, address, admission and discharge date, duration of hospital stay, and discharge status (D/C: normal discharged due to recovered or improved; expired: patients who died during hospital stay; signed and left (S/L): patients or family who signed on the statement that they did not want to receive additional treatment and left the hospital on their own will; and absconded (Abs): patients who left the hospital without giving notice to the health care providers). Second, clinical features, and other clinical information were extracted from the medical records; including stroke types as diagnosed by a physician, such as ‘ischemic’ or ‘haemorrhagic’ stroke (i.e. intra-cranial haemorrhage, ICH; or sub-arachnoid haemorrhage, SAH); and risk factors such as the patient’s medical histories of hypertension, diabetes mellitus, cardiovascular diseases, previous stroke or transient ischaemic attack (TIA), smoking habits, alcohol consumption, and betel chewing.

**Fig. 1:**
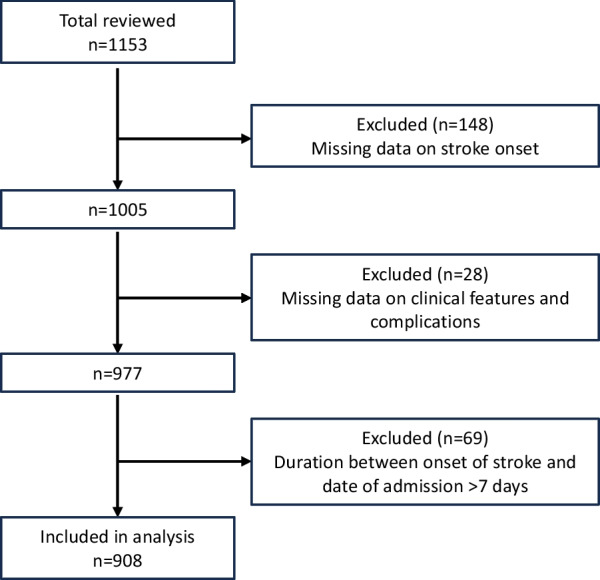
Flow diagram of the selection of stroke cases for inclusion in analysis

Medical records were extracted from 1153 stroke patients. Of these, 148 cases were excluded for having missing data on stroke onset, and 28 cases were excluded for lacking critical clinical features and complications such as GCS at the time of admission, fever during the hospital stay, and records of blood pressure measurement. An additional 69 cases were removed from analysis because the patients were admitted to the hospital more than 7 days after the stroke onset. The final analysis therefore focused on the data from 908 cases (Fig. 1).

### Statistical analysis

Categorical variables are described as a number count or percentage, and Chi-square test was used for analyses. Continuous variables are described either as median with interquartile range or mean with standard deviation; and analysed using the Mann–Whitney U test or Student’s t-test as appropriate.

For the outcome variable, patients were categorized into two groups which were improved (normal discharge) and unimproved (non-normal discharge). Improved patients were patients who were discharged from the hospital with good clinical outcome. Unimproved patients included expired cases, signed and leave (S/L) cases, and absconded (Abs) cases. S/L and Abs cases were considered unimproved because these patients or their family members chose to discharge themselves, even though their medical condition had not improved, and against the medical advice provided. A multiple logistic regression model was applied to identify factors associated with the unimproved stroke outcome. Confounding factors considered in the multiple logistic regression analyses included the type of stroke; history of hypertension, diabetes, and previous stroke; Glasgow Coma Scale (GCS) at the time of admission; and fever during the hospital stay. Stata (IC version 15.1) and Microsoft Excel (version 16.26) were used for data analysis.

## Results

### General characteristics of the stroke patients

**Fig. 2:**
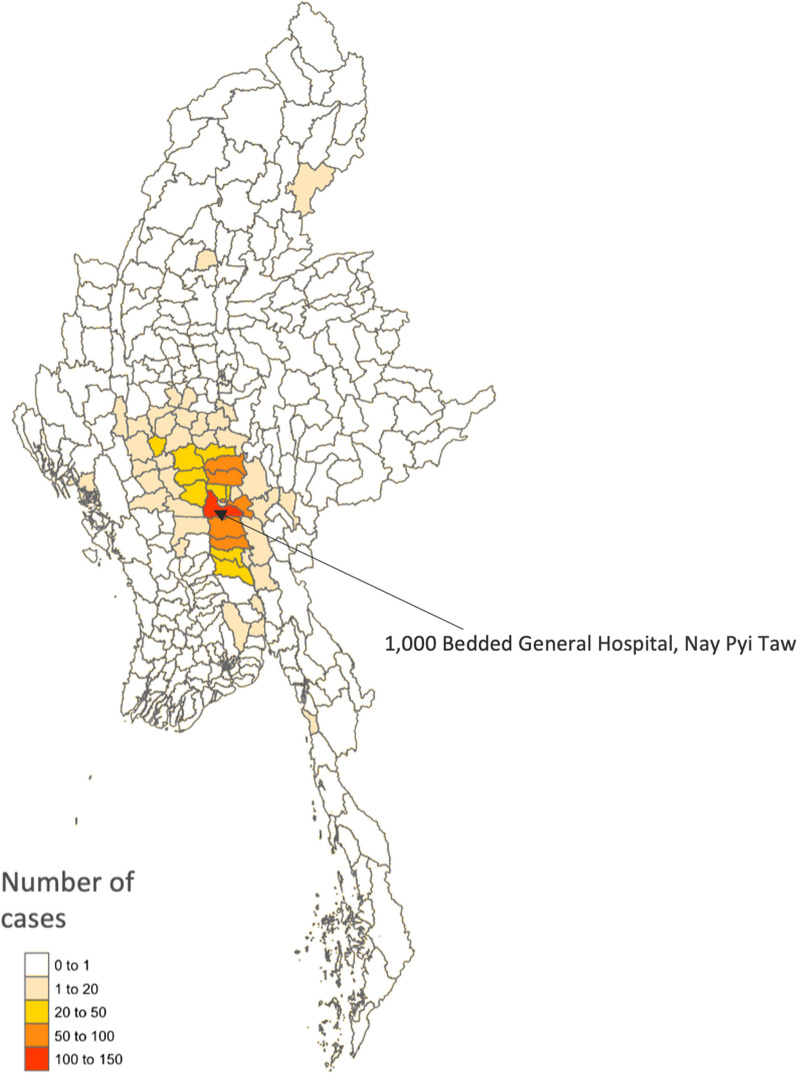
The residential location (township level) of admitted stroke cases admitted to the 1000 Bedded General Hospital, Nay Pyi Taw

**Fig. 3:**
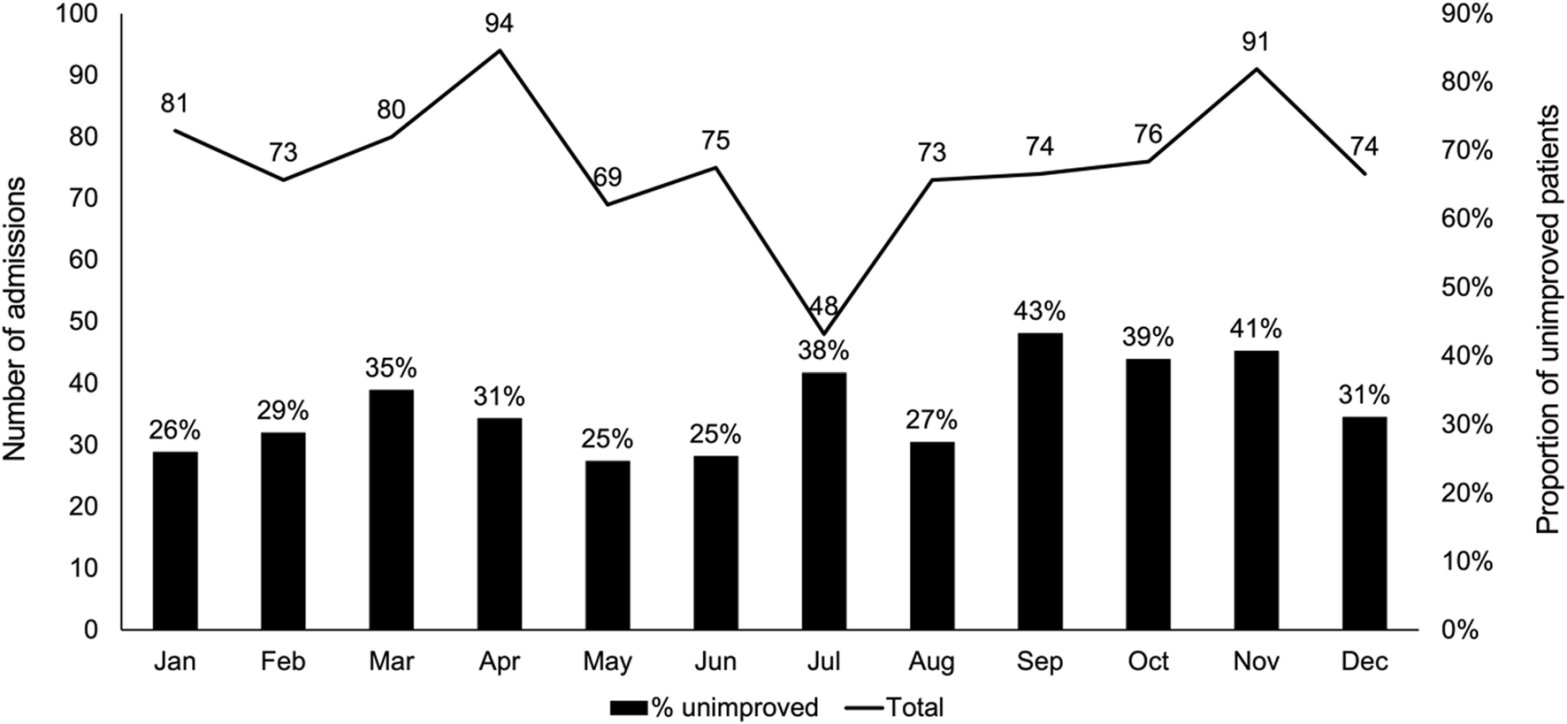
Number of monthly stroke admissions and proportion of unimproved of stroke patients in 1000 Bedded General Hospital, Nay Pyi Taw (2017)

The characteristics of the admitted stroke patients are outlined in Table 1 and indicate that a majority were males (61%), and resided in rural areas (66%). Referrals from other hospitals or health care facilities accounted for 33% of the stroke patients. The coverage area was wide, with patients originating from various regions, including Mandalay Region (46%) followed by Bago Region (18%), Magway Region (17%), Nay Pyi Taw Union Territory (17%) (Table 1 and Fig. 2). Figure 3 illustrates the distribution of the number of cases by month, and reveals seasonal fluctuations. April and November had the highest numbers of admissions; while July had the lowest. The predominant risk factors were hypertension (80%); with tobacco usage (26%) and regular alcohol consumption (21%) following as subsequent most prevalent risk factors (Table 1). Computed tomography (CT) scan results were documented in 94% of the cases, and encompassed 856 patients. Among the stroke patients, 49% were identified as haemorrhagic stroke patients, while 43% were diagnosed with ischaemic stroke. Improved outcome was observed in 67% of the patients.

**Table 1:** General characteristics of the stroke patients admitted to 1000 Bedded General Hospital, Nay Pyi Taw (2017) [*N* = 908]

Characteristics	Number	Percentage
Age group, years		
< 39	77	8.5
40–49	113	12.4
50–59	204	22.5
60–69	257	28.3
70–79	177	19.5
80 +	80	8.8
Sex		
Male	555	61.1
Female	353	38.9
Residence (*N* = 901)		
Urban	310	34.4
Rural	591	65.6
Origin of patient		
Mandalay Region	415	46.1
Bago Region	159	17.7
Magway Region	155	17.2
Nay Pyi Taw	149	16.5
Others	30	3.3
Risk factors		
Hypertension	726	80.0
Tobacco usage	236	26.0
Regular alcohol consumption	191	21.0
Diabetes mellitus	157	17.3
History of previous stroke or TIA	90	9.9
History of cardiovascular disease	56	6.2
Type of stroke		
Haemorrhagic	447	49.2
Ischaemic	392	43.2
Mixed	1	0.1
Not recorded	68	7.5
Outcome		
Improved	613	67.5
Unimproved	295	32.5

### Characteristics of improved versus unimproved stroke patients

Table 2 outlines the characteristics, risk factors, clinical features, and complications among improved and unimproved patients. The proportion of improved outcomes was significantly higher among those who were directly admitted to the hospital versus those referred from other health facilities (*p* = 0.02). Unimproved patients showed a higher prevalence of hypertension (*p* = 0.01), whereas a history of previous stroke or TIA and tobacco usage were more common risk factors among improved patients (*p* < 0.001 and *p* = 0.007). The improved patients also had lower admission blood pressure and blood sugar levels compared to unimproved patients (*p* < 0.001). The median Glasgow Coma Scale (GCS) score at the time of admission was 15 for improved patients and 8 for unimproved patients (*p* < 0.001). The presence of fever and aspiration pneumonia during the hospital stay was more common in unimproved patients (*p* < 0.001 and *p* = 0.02). Improved patients had longer hospital stays compared to their unimproved counterparts (*p* < 0.001).

**Table 2:** Characteristics, risk factors, clinical features, and complications among improved and unimproved patients admitted to 1000 Bedded General Hospital, Nay Pyi Taw (2017)

Characteristics	Improved (*n* = 613)	Unimproved (*n* = 295)	*p* value
Age: mean years ± SD	60.6 ± 14.2	60.2 ± 14.9	0.666
Sex (%)			
Male	375 (67.6)	180 (32.4)	0.964
Female	238 (67.4)	115 (32.6)	
Residence (%)*			
Urban	207 (66.8)	103 (33.2)	0.557
Rural	406 (68.0)	185 (31.3)	
Referral status (%)			
Direct admission	426 (70.2)	181 (29.8)	**0.015**
Referral from other health facility	187 (62.1)	114 (37.9)	
Duration of hospital stay: median days (IQR)	5 (3–9)	2 (1–5)	**< 0.001**
Risk factors (%)			
Hypertension	476 (77.7)	250 (84.8)	**0.012**
Diabetes mellitus	97 (15.8)	60 (20.3)	0.092
Tobacco usage	176 (28.7)	60 (20.3)	**0.007**
Regular alcohol drinking	130 (21.2)	61 (20.7)	0.855
Previous stroke/TIA	76 (12.4)	14 (4.8)	**< 0.001**
Cardiovascular diseases	44 (7.2)	12 (4.1)	0.068
Clinical features during admission			
SBP: mean mmHg ± SD	151 ± 28	162 ± 38	**< 0.001**
DBP: mean mmHg ± SD	91 ± 16	97 ± 22	**< 0.001**
GCS: median (IQR)	15 (13–15)	8 (4–11)	**< 0.001**
Blood sugar level: mean ± SD	136 ± 51	164 ± 57	**< 0.001**
Type of stroke (%)			
Haemorrhagic	239 (53.5)	208 (46.5)	**< 0.001**
Ischaemic	314 (80.1)	78 (19.9)	
Complications during hospitalization (%)			
Seizures	24 (3.9)	18 (6.1)	0.142
Fever	152 (24.8)	179 (60.7)	**< 0.001**
Aspiration pneumonia	24 (3.9)	22 (7.5)	**0.023**

*Improved (*n* = 613), unimproved (*n* = 288)

SBP: systolic blood pressure on admission; DBP: diastolic blood pressure on admission

### Risk factors association with unimproved stroke outcomes

Results from univariate logistic regression showed that haemorrhagic stroke patients were associated with an unimproved outcome (OR 3.50, 95% CI 2.57–4.78) (Table 3). Among the risk factors, hypertension was associated with an unimproved outcome (OR 1.60, 95% CI 1.10–2.31). Patients with an admission GCS lower than 14 were likely to have an unimproved outcome (OR 5.95, 95% CI3.95–8.94 for GCS 9–14; OR 58.40, 95% CI 35.20–97.00 for GCS < 9). Fever (OR 4.68, 95% CI 3.48–6.30) and aspiration pneumonia (OR 1.98, 95% CI 1.09–3.59) were two complications which were associated with an unimproved outcome. Patients were less likely to improve if they were referred from other health facilities (OR 1.43, 95% CI 1.07–1.92), had a systolic blood pressure greater than 150 mmHg at the time of admission (OR 1.46, 95% CI 1.09–1.95), or had a blood sugar level greater than 200 mg/dl at the time of admission (OR 1.54, 95% CI 1.10–2.14). Patients were more likely to improve if they had suffered a previous stroke/TIA (OR 0.35, 95% CI 0.20–0.63).

**Table 3:** Factors associated with unimprovement among stroke patients admitted to 1000 Bedded General Hospital, Nay Pyi Taw (2017)

Characteristics	Crude OR	95% CI	*p* value
Age			
< 40	1	Reference	
40–59	1.06	0.62–1.79	0.836
> 60	0.87	0.52–1.45	0.593
Sex			
Female	1	Reference	
Male	0.99	0.75–1.32	0.964
Residence			
Urban	1	Reference	
Rural	0.92	0.69–1.23	0.557
Referred from a health facility	1.43	1.07–1.92	**0.015**
Risk factors			
Hypertension	1.60	1.10–2.31	**0.013**
Diabetes mellitus	1.36	0.95–1.94	0.093
Tobacco use	0.63	0.45–0.88	**0.007**
Alcohol drinking	0.97	0.69–1.36	0.855
Previous stroke/TIA	0.35	0.20–0.63	**0.001**
Cardiovascular diseases	0.55	0.29–1.05	0.072
SBP (> 150 mmHg) during admission	1.46	1.09–1.95	**0.011**
DBP (> 90 mmHg) during admission	1.27	0.94–1.73	0.119
BS on admission (> 200 mg/dl)	1.54	1.10–2.14	**0.010**
GCS at the time of admission			
15–14	1	Reference	
13–9	5.95	3.95–8.94	**< 0.001**
$\le$ 8	58.4	35.2–97.0	**< 0.001**
Type of stroke			
Ischaemic	1	Reference	
Haemorrhagic	3.50	2.57–4.78	**< 0.001**
Complications during hospitalization			
Seizure	1.59	0.85–2.99	0.145
Fever	4.68	3.48–6.30	**< 0.001**
Aspiration pneumonia	1.98	1.09–3.59	**0.025**

SBP: systolic blood pressure; DBP: diastolic blood pressure; BS: blood sugar level during admission

**Fig. 4:**
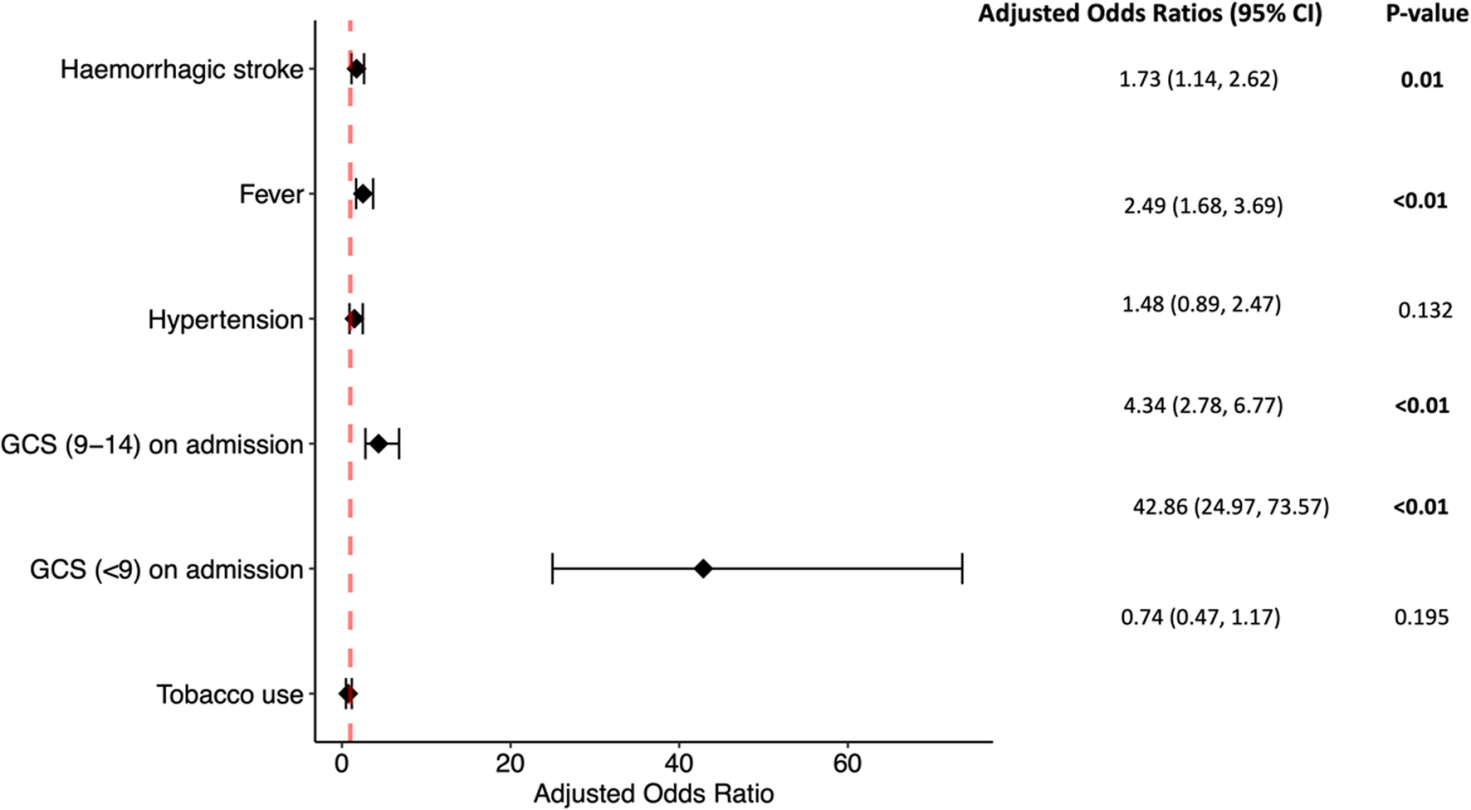
Multivariate analysis of selected factors associated with unimproved outcome

The multivariate analysis indicated that poor prognoses were associated with haemorrhagic stroke (adjusted odd ratios: AOR 1.73, 95% CI 1.14–2.62), development of fever during the hospital stay (AOR 2.49, 95% CI 1.67–3.69), and lower GCS levels (AOR 4.34, 95% CI 2.78–6.77 for GCS 9–14 and AOR 42.86, 95% CI 24.97–73.57 for GCS < 9) (Fig. 4). Hypertension was associated with unimproved outcome in bivariate analysis; however, this significance was not observed in multivariate analysis (AOR 1.48, 95% CI 0.89–2.47) (Fig. 4).

## Discussion

In this study, we assessed the stroke types, risk factors, clinical features, and outcomes among hospitalized patients in Myanmar. Haemorrhagic stroke was found to be the most prevalent type (49%), contrary to studies in other countries where ischaemic stroke was reported as more common [6, 9–12]. This difference could be due to a selection bias of the study setting; specifically, the Nay Pyi Taw General Hospital is one of four public hospitals in the country with neurology departments and neurosurgical departments for the optimal care of haemorrhagic stroke cases. In Myanmar, stroke cases are typically managed by internists in urban areas and general practitioners in rural areas [13]. However, when neurosurgical interventions are necessary for haemorrhagic stroke patients, they are referred to hospital which have a specialized department. This could potentially contribute to the higher proportion of haemorrhagic stroke cases in our study. Despite this variation, the findings of the analysis on poor prognosis remain valid.

Among the admissions for stroke, hypertension was the most common risk factor. The prevalence of hypertension among the stroke cases was one of the highest among South, East and South-East Asia [5]. For acute stroke, hypertension is a major risk factor and thus maintaining optimal blood pressure during the management of stroke is important for the outcome [14].

The median duration of hospital stay was 4 days which was similar to the result of a study done in this hospital in 2016 [15], as well as the results from a study of stroke epidemiology in Thailand [9]; but shorter than observed in a study in Ethiopia [12]. There was no significant difference between the duration of hospital stay in ischaemic versus haemorrhagic stroke (Additional file 1: Table S1). The duration of hospital stays among the improved patients was longer than the patients who were not improved. S/L and absconded cases were classified as unimproved cases in the analysis, and this may be one of the reasons of longer hospital stays among the improved patients. The proportion of the S/L (27%) (Additional file 1: Table S1) were higher than that of 2016 (15%) [15]. No health insurance system exits in Myanmar, and the out-of-pocket (OOP) payment for the health care services is high [16]. As the duration of hospital stays became longer, opportunity costs such as absence from work may be reasons underlying S/L or absconded cases. In this study, we were not able to include the income status of the stroke patients and thus it was not possible to assess the income status of the S/L and absconded cases.

Among the stroke patients, 10% had a history of a previous stroke or TIA, and they were more commonly admitted with ischaemic strokes (Additional file 1: Table S1). Notably, these cases exhibited better outcome. Studies have shown that 7.4% of TIA patients develop a stroke within 90 days of the attack [17]. Seeking consultation with physicians during TIA may have contributed to the improved outcomes observed in these cases.

Of haemorrhagic stroke cases, most were ICH and 46% were poor prognosis. This finding aligns with the findings of Andersen and colleagues, which reported an increased severity and a greater risk of death in patients with haemorrhagic strokes compared to those with ischaemic stroke [18]. Another study also reported that the case fatality of ICH was 40% at one month and 54% at one year, which is also consistent with our finding [19].

Conscious level at the admission assessed by GCS was a good predictor of prognosis in this study, as it has been shown elsewhere [20]. In our study, admission GCS among improved patients was significantly higher than admission GCS among unimproved patients. This finding was compatible with studies done in Nigeria [10, 21]. This may be due to the nature of the disease itself, as haemorrhage causes increased intracranial pressure by haematoma, perihematomal oedema, and intraventricular extension [22].

Having fever was revealed as a predictor of poor prognosis. This finding was compatible with the result of meta-analysis which reported fever as consistent association with worse outcome whether of ischaemic or haemorrhagic stroke [23]. Fever is a sign of infection, and it is known that stroke patients frequently experience infections. The main causes of infection are aspiration pneumonia, and infections caused by common commensal bacteria due to bacterial translocation [24]. Therefore, consolidation in infection control including appropriate application of antibiotics could be a possible contributor to improve the prognosis. This policy is not currently integrated into stroke management in Myanmar; and therefore, it should be considered if such a standardized management procedure should be established.

This study has some limitations. Firstly, the study population may not be representative of all stroke cases in Myanmar, as the data in this study were based on a single tertiary hospital. Secondly, the timeframe of the study was confined to one year (2017), and having longitudinal data may be more informatic. Thirdly, we could not follow up on patients following discharge from the hospital; and therefore, their long-term prognosis was unknown. However, it is unlikely that many of the S/L or Abs cases improved without appropriate hospital care management. Finally, we were not able to obtain information on other modifiable risk factors such as lifestyle or dietary habits, which may have influenced the outcomes of the study.

## Conclusion

Stroke is more common among the older age group population in Myanmar, and haemorrhagic stroke is the most common type among the hospitalized stroke patients in the hospital under the study. Hypertension is the most common risk factor for both ischaemic and haemorrhagic strokes. Multi-centre and multi-year studies should be conducted for a better understanding of stroke epidemiology in Myanmar. It is important to raise public awareness on the risk factors and the prevention measures of strokes. Moreover, research exploring access to healthcare services, quality of stroke care and socioeconomic factors would provide valuable insights for developing targeted interventions aimed at stroke prevention and management. It is also important to increase the number of stroke care units to cover the wider geographical population of Myanmar.

### Supplementary Information

**Additional file 1: Table S1. **Characteristics, risk factors, clinical features and complications among ischaemic stroke and haemorrhagic stroke.

## Data Availability

The dataset used in this study was collected with the permission of the government of Myanmar; and therefore it is not publicly available.

## Abbreviations

Abs: Absconded
AOR: Adjusted odds ratio
CAMRS: Computer assisted medical record system
CT Scan: Computed tomography scan
D/C: Normal discharged (improved)
DALY: Disability-adjusted life year
DBP: Diastolic blood pressure
DM: Diabetes mellitus
GCS: Glasgow Coma Scale
ICD: International Classification of Diseases
ICH: Intracerebral haemorrhagic
OR: Odds ratio
S/L: Signed and left
SAH: Subarachnoid haemorrhagic
SBP: Systolic blood pressure
TIA: Transient ischaemic attack
WHO: World Health Organization
