# Supplementary material for: A descriptive study of stroke types, risk factors, clinical features, and outcomes in a tertiary hospital in Myanmar
Source: Trop Med Health. 2024 Mar 18;52:26. doi: 10.1186/s41182-024-00592-6 (PMC10946113; doi:10.1186/s41182-024-00592-6)
Supplement: Supplementary file 1 — Additional file 1: Table S1. Characteristics, risk factors, clinical features and complications among ischaemic stroke and haemorrhagic stroke. [file 41182_2024_592_MOESM1_ESM.docx]

**Stroke types, risk factors, clinical features and outcomes**

**in a tertiary hospital, Myanma****r, a descriptive study**

**Authors:**

Thant Zin Tun ^1, 2^, Su Myat Han ^1, 3^, Kazuhiko Moji ^1^, Mitsuaki Matsui ^1, 4^

**Affiliations:**

1 Department of Global Health, Nagasaki University School of Tropical Medicine and Global Health, Nagasaki, Japan

2 Graduate School of Biomedical Sciences, Nagasaki University, Nagasaki, Japan

3 Department of Infectious Disease Epidemiology, Faculty of Epidemiology and Population Health, London School of Hygiene and Tropical Medicine, London, UK,

4 Department of Public Health, Kobe University Graduate School of Health Sciences, Kobe, Japan

**Corresponding author:**

Thant Zin Tun

Graduate School of Biomedical Sciences, Nagasaki University, Nagasaki, Japan

Department of Protozoology, Institute of Tropical Medicine, Nagasaki University, Nagasaki, Japan

E-mail: [dr.thantzintun90@gmail.com](mailto:dr.thantzintun90@gmail.com)

Phone: +81 958197838

Supplementary Table 1: Characteristics, risk factors, clinical features and complications among ischaemic stroke and haemorrhagic stroke

| General Characteristics | Ischaemic (n=392) | Haemorrhagic (n=447) |
| --- | --- | --- |
| Age: mean years ± SD | 62.4± 13.9 | 58.5 ± 14.8 |
| **Sex** |  |  |
| Male | 228 (58.2%) | 288 (64.4%) |
| Female | 164 (41.8%) | 159 (35.6%) |
| **Residence** |  |  |
| Urban | 137 (35.1%) | 144 (32.6%) |
| Rural | 253 (64.9%) | 298 (67.4%) |
| Referred from health facilities | 95 (24.2%) | 192 (43%) |
| Duration of hospital stayed: median (IQR) | 4 (2 - 9) | 4 (2 - 8) |
| **Risk Factors** |  |  |
| Hypertension | 295 (75.3%) | 376 (84.1%) |
| Diabetes Mellitus | 73 (18.6%) | 69 (15.4%) |
| Tobacco usage | 113 (28.8%) | 103 (23.0%) |
| Alcohol | 82 (20.9%) | 95 (21.3%) |
| Previous stroke/TIA | 57 (14.5%) | 16 (3.6%) |
| Cardiovascular diseases | 37 (9.4%) | 10 (2.2%) |
| **Clinical Features** |  |  |
| SBP: Mean ± SD | 146 ± 28 | 163 ± 34 |
| DBP: Mean  ± SD | 89 ± 17 | 97 ± 19 |
| GCS: median (IQR) | 15 (11 - 15) | 11 (7 - 15) |
| Blood sugar level:  mean mg/dl ± SD | 140 ± 57 | 151 ± 52 |
| **Complications** |  |  |
| Seizures | 19 (4.9%) | 21 (4.7%) |
| Fever | 100 (25.5%) | 221(49.4%) |
| Aspiration pneumonia | 23 (5.9%) | 17 (3.8%) |
| **Discharge status** |  |  |
| Normal discharge | 314 (80.1%) | 239 (53.5%) |
| Expired | 10 (2.6%) | 41 (9.2%) |
| Absconded (Abs) | 3 (0.7%) | 5 (1.1%) |
| Sign and left (S/L) | 65 (16.5%) | 162 (36.2%) |
